# Oral Sucrosomial Iron Is as Effective as Intravenous Ferric Carboxy-Maltose in Treating Anemia in Patients with Ulcerative Colitis

**DOI:** 10.3390/nu13020608

**Published:** 2021-02-12

**Authors:** Lorenzo Bertani, Domenico Tricò, Federico Zanzi, Giovanni Baiano Svizzero, Francesca Coppini, Nicola de Bortoli, Massimo Bellini, Luca Antonioli, Corrado Blandizzi, Santino Marchi

**Affiliations:** 1 Department of Translational Research and New Technologies in Medicine and Surgery, University of Pisa, 56100 Pisa, Italy; federico.zanzi@outlook.com (F.Z.); gbaianosvizzero@gmail.com (G.B.S.); coppini.francesca@gmail.com (F.C.); nicola.debortoli@unipi.it (N.d.B.); mbellini58@gmail.com (M.B.); santino.marchi@unipi.it (S.M.); 2 Department of Surgical, Medical and Molecular Pathology and Critical Care Medicine, University of Pisa, 56100 Pisa, Italy; domenico.trico@unipi.it; 3 Department of Clinical and Experimental Medicine, University of Pisa, 56100 Pisa, Italy; lucaant@gmail.com (L.A.); c.blandizzi@gmail.com (C.B.)

**Keywords:** anemia, iron, ulcerative colitis, clinical

## Abstract

Anemia is a frequent complication of ulcerative colitis, and is frequently caused by iron deficiency. Oral iron supplementation displays high rates of gastrointestinal adverse effects. However, the formulation of sucrosomial iron (SI) has shown higher tolerability. We performed a prospective study to compare the effectiveness and tolerability of oral SI and intravenous ferric carboxy-maltose (FCM) in patients with ulcerative colitis in remission and mild-to-moderate anemia. Patients were randomized 1:1 to receive 60 mg/day for 8 weeks and then 30 mg/day for 4 weeks of oral SI or intravenous 1000 mg of FCM at baseline. Hemoglobin and serum levels of iron and ferritin were assessed after 4, 8, and 12 weeks from baseline. Hemoglobin and serum iron increased in both groups after 4 weeks of therapy, and remained stable during follow up, without significant treatment or treatment-by-time interactions (*p* = 0.25 and *p* = 0.46 for hemoglobin, respectively; *p* = 0.25 and *p* = 0.26 for iron, respectively). Serum ferritin did not increase over time during SI supplementation, while it increased in patients treated with FCM (treatment effect, *p* = 0.0004; treatment-by-time interaction effect, *p* = 0.0002). Overall, this study showed that SI and FCM displayed similar effectiveness and tolerability for treatment of mild-to-moderate anemia in patients with ulcerative colitis under remission.

## 1. Introduction

Ulcerative colitis (UC) is a chronic inflammatory disease involving the colon and rectum [1]. Despite the inflammation is limited to the colonic mucosa, systemic complications, are common in patients with UC [2]. Anemia is one of the most common complications of UC, and its prevalence ranges between 6% and 70%, depending on the evaluated population: Patients experiencing a flare, or hospitalized, or evaluated at diagnosis display higher rates of anemia, which decreases during the first 4 years after the disease onset and remains stable thereafter [3,4,5]. A recent population-based cohort study showed that the incidence of anemia in UC was 12.9 per person-years, with a prevalence of 16.5% [6]. Moreover, a European population-based inception cohort study showed that 39% of UC patients experienced at least one episode of anemia during the first 12 months after diagnosis [7]. In the majority of cases, anemia in UC is related to the combination of chronic iron deficiency (iron deficiency anemia) and the condition of systemic inflammation (anemia of chronic diseases) [8].

Iron supplementation is recommended in all patients with inflammatory bowel disease (IBD) and iron deficiency anemia. In this regard, randomized controlled trials [9,10] and real-world studies [11] showed that intravenous ferric carboxy-maltose (FCM) is effective for treatment of anemia in UC. Its use has increased progressively in recent years, since it displayed a lower rate of infusion reactions as compared with other formulations of intravenous iron [12]. However, intravenous formulations are not well accepted by all patients and are burdened by higher costs for health care systems. Accordingly, current guidelines suggest that intravenous iron should be used as first line therapy only in patients with active disease, moderate-to-severe anemia and intolerance to oral iron [13]. Intolerance to oral iron is a frequent issue in UC, since about 25% of patients with anemia withdraw from oral iron treatment due to the occurrence of gastrointestinal symptoms (abdominal pain, diarrhea, dyspepsia) [14]. Sucrosomial^®^ Iron (SI) is an innovative oral iron formulation, in which ferric pyrophosphate is encapsulated in a phospholipid bilayer plus a sucrester matrix, which protect the digestive mucosa from direct contacts with iron and facilitate its absorption through para-cellular and trans-cellular routes [15]. These features confer high bioavailability and excellent gastrointestinal tolerance to this oral formulation.

Although oral supplementation with SI was found effective in treating iron deficiency anemia in patients with UC [16], a direct comparison between SI and FCM has been never performed. Therefore, the aim of this prospective study was to compare the efficacy and safety of treatments with oral SI and intravenous FCM in the management of iron deficiency anemia in patients with UC under clinical, endoscopic and biochemical remission.

## 2. Methods

We performed a prospective, randomized, open-label study at the Gastroenterology Unit of Pisa University Hospital (Pisa, Italy).

From May to July 2020 we recruited consecutive patients, who: (1) Were affected by UC in remission; (2) were 18-year older; (3) had mild-to-moderate anemia related to iron deficiency; (4) agreed to sign the informed consent to participate in the study.

The diagnosis of UC had been confirmed previously by histology. Remission was defined at baseline as a Partial Mayo Score <2, with C-Reactive Protein levels <0.5 mg/dL and fecal calprotectin levels <150 mg/kg in the week preceding treatment initiation [17]. Moreover, all the included patients had performed a colonoscopy, showing a Mayo Endoscopic Score <2, in the six months preceding their enrolment.

We included patients with mild-to-moderate anemia according to the World Health Organization criteria [18]. For inclusion in the study, hemoglobin (Hb) levels had to range from 8 to 13 g/dL, considering that all patients were adults living at the sea level. Moreover, to define the iron deficiency as the main cause of anemia, iron levels had to be lower than 70 μg/dL. Serum ferritin levels had to be lower than 100 ng/mL.

Patients were randomized 1:1 into two groups using a computer-generated random number list: (1) Treatment with FCM 1000 mg, one infusion i.v. at baseline; (2) treatment with SI 60 mg q.d. for 8 weeks since baseline and then 30 mg q.d. for 4 weeks. All the included patients had a body weight <70 kg, in order to avoid biases related to the different dosage required for FCM in case of subjects with body weight higher than 70 kg [12].

A complete blood count (to evaluate Hb levels), serum iron and serum ferritin were measured at baseline and then after 4, 8, and 12 weeks in all participants by laboratory technicians, who were blind to patient treatments. All assessments were performed at the same laboratory, using DASIT XE-5000/2100 kit (Dasitgroup, Milan, Italy) for the assay of Hb and Roche Cobas 6000 and Cobas Modul E kits (Roche Italia, Monza, Italy), for the evaluation of iron and ferritin, respectively. At each time-point, a team of clinicians performed a complete clinical visit with physical examination, and the possible presence of adverse events typically related to iron therapy (such as bloating, nausea, diarrhea, and constipation) were recorded. At every time-point, the patients allocated in the SI cohort had to return the blisters, in order to check for their compliance. Patients experiencing a disease flare during the follow-up were excluded from the analysis; disease flare was defined as an increase in at least two points of the Partial Mayo Score.

The present study was conducted in full compliance with the Declaration of Helsinki and was approved by the Ethical Committee of Pisa University Hospital (CEAVNO) on 30 April 2020, with the protocol number 17142. All the included patients provided their informed consent.

### Statistical Analysis

The primary endpoint was to evaluate whether Hb levels differed in the two treatment groups. As secondary endpoints, we evaluated possible differences in serum iron and ferritin levels, as well as the occurrence of gastrointestinal symptoms. A sample size of 32 subjects (*n* = 16 per group) was calculated to provide at least 90% power to detect a significant treatment-by-time interaction effect for Hb (partial η^2^ = 0.06). A total of 42 subjects were enrolled allowing for a 30% drop out rate.

Baseline group differences were tested using Mann–Whitney U test or Fisher exact test. Repeated measures were analyzed using two-way repeated measures ANOVA including “treatment” or “sex”, “time”, and their interaction as independent variables. Post-hoc multiple comparisons were corrected using Dunn–Šidák correction. Correlations were tested using Spearman correlation. Subgroup analyses by sex were performed to examine potential differences in females and males.

Analyses were performed using JMP Pro 14.2.1 (SAS, Cary, NC, USA) at a two-sided α level of 0.05. The sample size calculation was performed using G*Power 3.1 [19].

## 3. Results

We enrolled 42 patients: 21 treated with FCM and 21 treated with SI, according to the study design. Two patients (one treated with FCM and one with SI) were excluded from the analysis, since they experienced a clinical relapse of UC during the follow-up. Therefore, 40 patients completed the follow-up and were included in the analysis (20 treated with FCM and 20 with SI). The main participants’ characteristics are shown in Table 1. They were matched for age and sex. There were also no differences in Hb, iron or ferritin levels at baseline.

No extra-intestinal symptoms were recorded in both groups. Both interventions were well tolerated, although oral iron supplementation was associated with transient mild gastrointestinal symptoms (bloating and flatulence) in one patient (5% vs. 0% incidence in the FCM group, *p* > 0.99). These symptoms were reported only during the first 8 weeks of treatment, when the dosage of SI was 60 mg, and disappeared when the dosage was tapered to 30 mg. In support of this good tolerance, the compliance was extremely high: 18/20 patients treated with SI displayed a compliance of 100%; one patient had 97.6% (he forgot to take 2 pills), and even the patient who reported the gastrointestinal symptoms had a compliance of 94%.

**Figure 1:**
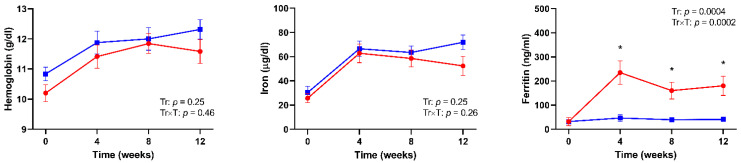
Time-trends of hemoglobin, serum iron, and serum ferritin levels in patients with ulcerative colitis (UC) treated with intravenous ferric carboxy-maltose (red lines) or oral sucrosomial^®^ iron (blue lines). *p* values for time (T) and treatment by time interaction (Tr × T) effects are shown.

The time-trends of Hb, iron, and ferritin levels are shown in Figure 1. Blood Hb levels increased on average by 1.1 g/dl (+11%) after 4 weeks of therapy in both groups and remained stable during follow up, without significant treatment or treatment-by-time interaction effects (*p* = 0.25 and *p* = 0.46, respectively). Paralleling the time course of Hb, serum iron levels increased on average by 37 μg/dl (+278%) and reached a plateau after 4 weeks of either therapy, without significant differences by treatment (treatment effect, *p* = 0.25; treatment-by-time interaction effect, *p* = 0.26). However, in the FCM group both Hb and iron showed a trend towards a decrease from 8 to 12 weeks of therapy. As expected, the percent increase in iron levels correlated positively with the increase in Hb levels (r = 0.34, *p* = 0.03). Serum ferritin levels did not increase over time during SI supplementation, whereas it increased on average by 203 ng/mL (+2050%) in patients treated with FCM, showing a peak at 4 weeks followed by a progressive decline (treatment effect, *p* = 0.0004; treatment-by-time interaction effect, *p* = 0.0002).

In subgroup analyses by sex, oral and intravenous iron supplementation yielded similar increases in Hb and iron levels in both females and males (Figure S1). Serum ferritin rise was numerically greater in females than males; however, sex differences did not reach statistical significance (sex effect, *p* = 0.24; sex by time interaction effect, *p* = 0.0002).

## 4. Discussion

This prospective study was designed to compare the effectiveness and gastrointestinal safety of two iron supplementation medications for anemia in UC. Our results show that FCM and SI induced a similar increase in Hb and serum iron levels, but FCM was more effective in increasing serum ferritin levels, without significant gender differences. In addition, both treatments were well tolerated and SI was associated with a very high patient compliance.

Although iron is commonly prescribed in UC patients, the optimal method for supplementation is currently under debate [14]. Intravenous formulations of iron were demonstrated to be effective, and the use of FCM instead of intravenous iron sucrose or ferrous sulfate has been associated with a reduction of infusion reactions [12]. In terms of efficacy, FCM was proven to be superior to iron sucrose and non-inferior to ferrous sulfate in increasing Hb levels at week 12 in patients with IBD [9,10]. However, despite its higher tolerability in comparison with other intravenous formulations, the discomfort often experienced with the intravenous administration compares very unfavorably with the easiness and high acceptance of oral therapy. Notably, there are also significant cost implications in choosing intravenous iron [14].

Oral formulations are useful in increasing Hb levels in UC both in adult [20] and children populations [21]. Moreover, the efficacy of oral iron has been demonstrated also in pregnant women with UC [22] and geriatric populations [23]. However, adverse digestive effects of iron oral formulations are common and have a significant impact on patients’ compliance. They are typically dose-dependent and more frequent in case of active disease, since the absorption of iron in the gastrointestinal tract is limited and, in the setting of UC, the ulcerated bowel mucosa undergoes exposure to unabsorbed iron [24]. For these reasons, current guidelines suggest a daily dosage of oral iron <100 mg, preferably in patients without active severe disease [24]. Nevertheless, in recent years a progressive shift from oral to intravenous iron supplementation has been observed [25]. In this context, SI has been shown to be effective in increasing Hb, iron and ferritin levels in patients with UC, who were intolerant to other oral formulations [26]. Accordingly, several experts recommend oral iron formulations with improved tolerability, such as SI, as a viable alternative to intravenous iron [27]. Conversely, intravenous iron should be preferred in patients intolerant to oral iron, who have active disease and moderate-to-severe anemia [13].

Several trials have been conducted to compare the effectiveness of oral and intravenous iron formulations in patients with IBD. However, the majority of studies evaluated oral iron sulphate, and thus the outcomes of oral therapy were particularly unsatisfactory. Lindgren et al. [28] compared intravenous iron sucrose and oral iron sulphate, showing that only 48% of patients tolerated the prescribed oral dose, and 52% reduced the dose or withdrew from treatment because of poor tolerance. Moreover, treatment with intravenous iron sucrose increased ferritin levels faster and more effectively than oral iron [28]. Of note, oral iron sulphate displayed poor results even in terms of effectiveness in comparison with parenteral iron, as demonstrated also in a Korean retrospective study [29]. Furthermore, as expected, when comparing intravenous iron sucrose with oral iron sulphate, the results in terms of side effects were significantly better for intravenous formulation [30]. With regard for oral ferrous fumarate, Erichsen et al. [31] showed that it could increase the clinical disease activity, at variance with intravenous iron sucrose, which increased only the intravascular oxidative stress, reducing the concentrations of plasma malondialdehyde and other antioxidants. However, even the increase in disease activity shown in oral iron therapies could be related to oxidative stress, although induced in gut lumen by the modulation of intestinal microbiota due to dietary iron supplementation [32].

Based on current data, the role of oral iron supplementation seems to be fairly inferior, when compared with intravenous therapy. However, no previous studies have compared oral SI with intravenous formulations in patients with IBD. Our favorable results could be explained by the unique structure of SI, where the protection of ferric pyrophosphate by a phospholipid bilayer plus a sucrester matrix (sucrosome) confers a significantly higher digestive tolerability [15]. Indeed, the sucrosome prevents the gastric inactivation and allows iron to be carried through the intestinal wall, thus reducing the risk of adverse effects related to the interaction of iron with the intestinal mucosa [33]. Moreover, this feature increases the iron absorption leading to a higher bioavailability, as compared with other iron salts [33].

Our results showed that SI was not as effective as FCM in increasing serum ferritin levels. This could be related to the different kinetics of iron dispensed: In intravenous therapy, 1000 mg were administered at baseline, increasing significantly iron levels and inducing the storage in the form of ferritin; conversely, with SI therapy we have provided 60 mg daily, which could be adequate only for the synthesis of hemoglobin in bone marrow. To confirm this, an in-vitro and ex-vivo experiment demonstrated how SI content was significantly higher in bone marrow after five hours after the administration [34].

The present study has some limitations. (1) The number of included patients is limited, and a higher sample size could be more appropriate to draw more definitive conclusions. However, we included more patients than those required to identify clinically relevant differences on the basis of the statistical power plan, and thus our results should be considered as reliable in nature. (2) Despite iron deficiency anemia is more frequent in Crohn’s disease, as compared with UC, patients with Crohn’s disease were not evaluated in our study. However, it is noteworthy that a chronic inflammation of small bowel, even though under remission, would likely interfere with the absorption of oral iron [35]. Therefore, we preferred to exclude patients with Crohn’s disease to obtain a homogenous patients’ cohort. (3) Dietary intake could modify iron bioavailability, although not to a significant extent in 12 weeks [36]. We suggested a typical Mediterranean diet to all the included patients. Nevertheless, we acknowledge that the collection of a food diary from every patient could have strengthened the significance of our results. (4) A double-blind design would be more convincing, especially with regard for the collection of side effects. (5) Hemoglobin and ferritin levels were not statistically different between groups both at baseline and in repeated-measure analysis; it should be noted however that they were numerically higher in the SI than FCM group (*p* ≤ 10). (6) The collection of other parameters of iron metabolism, such as serum transferrin, transferrin saturation, or hepcidin, would have increased the significance of our results. However, the present trial should be intended as a pilot, explorative study, able to pave the way for the use of SI in larger cohort of patients with UC.

In the present study, the major point of strength is the putative impact that the results could have in the clinical practice, if confirmed in larger cohorts: The similar efficacy of SI and FCM could support the preferential choice of oral therapy, with important savings for health-care expenditure. Furthermore, differently from other previous studies with oral iron, SI showed a remarkable tolerability, which lead to an extremely high compliance and an absolute lack of important side effects in our cohort. Another important point of strength is the study design. Indeed, the high stringency of the inclusion and exclusion criteria allowed us to select an extremely homogenous patients’ population, and this avoided selection biases. Moreover, the two cohorts were comparable at baseline in terms of Hb, iron and ferritin levels, thus justifying our conclusions.

## 5. Conclusions

The present study showed a similar effectiveness of SI and FCM in the management of anemia in patients with UC under remission. Conversely, iron stores, as expressed by ferritin levels, were significantly higher in patients treated with FCM. No significant gastrointestinal adverse effects were recorded in our treatment cohorts. Overall, this study suggests the preferential use of oral supplementation with SI in patients with UC under remission, since its benefits in terms of effectiveness and tolerability combine favorably with its lower cost and elevated patient acceptance.

## Figures and Tables

**Table 1:** Characteristics of the study participants treated with ferric carboxy-maltose (FCM) or sucrosomial^®^ iron (SI).

	FCM	SI	*p*
Number	20	20	-
Age (years)	43.5 [31.5–66.8]	42.0 [29.5–60.8]	0.65
Sex (*n*, %)			0.99
Men	12 (60.0)	11 (55.0)	
Women	8 (40.0)	9 (65.0)	
Hemoglobin (g/dl)			
Baseline	10.3 [9.0–11.0]	11.1 [9.9–11.6]	0.10
12 weeks	11.8 [10.7–12.7]	12.2 [11.5–13.3]	0.17
Mean change from baseline	1.3 [0.7–2.2]	1.1 [0.4–2.1]	0.56
Iron (μg/dl)			
Baseline	22 [14–38]	23 [12–39]	0.78
12 weeks	36 [26–72]	71 [50–97]	0.03
Mean change from baseline	26 [9–60]	47 [7–56]	0.51
Ferritin (ng/mL)			
Baseline	10 [5–13]	16 [9–25]	0.09
12 weeks	131 [90–225]	26 [15–44]	0.001
Mean change from baseline	137 [61–231]	9 [3–17]	<0.0001

Mean changes from baseline were calculated as the difference between average levels at week 4, 8, and 12 and baseline levels.

## Data Availability

Data are available upon request to corresponding author.

## Supplementary Materials

The following are available online at https://www.mdpi.com/2072-6643/13/2/608/s1, Figure S1: Time-trends of Hb, serum iron, and serum ferritin levels in women (top panels) and men (bottom panels) with UC treated with intravenous ferric carboxy-maltose (red lines) or oral sucrosomial^®^ iron (blue lines).
