# Supplementary material for: Oral Sucrosomial Iron Is as Effective as Intravenous Ferric Carboxy-Maltose in Treating Anemia in Patients with Ulcerative Colitis
Source: Nutrients. 2021 Feb 12;13(2):608. doi: 10.3390/nu13020608 (PMC7917674; doi:10.3390/nu13020608)
Supplement: Supplementary file 1 [file nutrients-13-00608-s001.pdf]

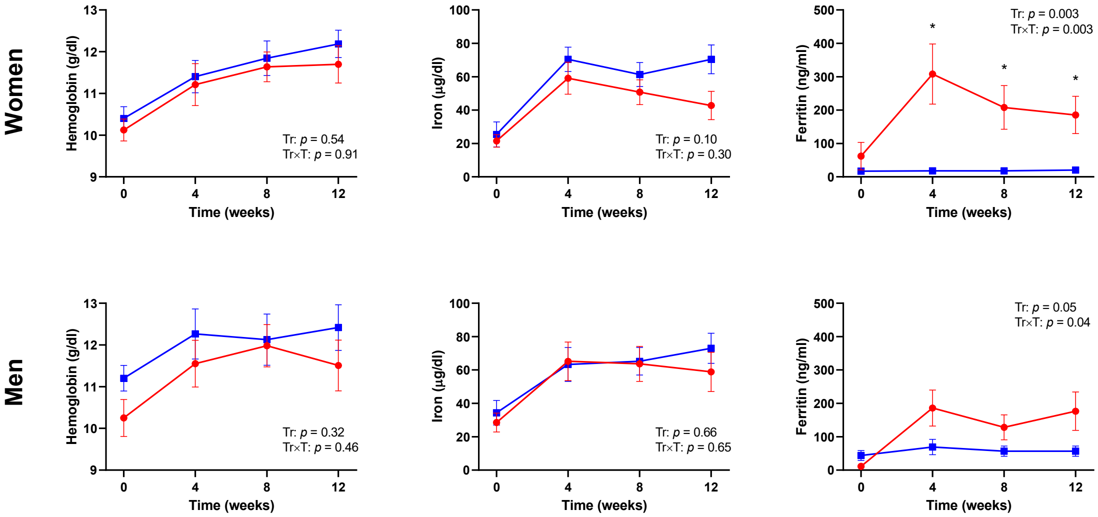


**Figure S1.** Time-trends of Hb, serum iron, and serum ferritin levels in women (top panels) and men (bottom panels) with UC treated with intravenous ferric carboxy-maltose (red lines) or oral sucrosomial® iron (blue lines).
